# Fine-Scale Phylogeographic Structure of *Borrelia lusitaniae* Revealed by Multilocus Sequence Typing

**DOI:** 10.1371/journal.pone.0004002

**Published:** 2008-12-23

**Authors:** Liliana R. Vitorino, Gabriele Margos, Edward J. Feil, Margarida Collares-Pereira, Libia Zé-Zé, Klaus Kurtenbach

**Affiliations:** 1 Departamento de Biologia Vegetal/Centro de Genética e Biologia Molecular, Faculdade de Ciências, Universidade de Lisboa, Campo Grande, Lisboa, Portugal; 2 Department of Biology and Biochemistry, University of Bath, Bath, United Kingdom; 3 Unidade de Leptospirose e Borreliose de Lyme, Instituto de Higiene e Medicina Tropical, Universidade Nova de Lisboa, Lisboa, Portugal; 4 Centro de Estudos de Vectores e Doenças Infecciosas, Instituto Nacional de Saúde Dr. Ricardo Jorge, Lisboa, Portugal; Centre for DNA Fingerprinting and Diagnostics, India

## Abstract

*Borrelia lusitaniae* is an Old World species of the Lyme borreliosis (LB) group of tick-borne spirochetes and prevails mainly in countries around the Mediterranean Basin. Lizards of the family *Lacertidae* have been identified as reservoir hosts of *B. lusitaniae*. These reptiles are highly structured geographically, indicating limited migration. In order to examine whether host geographic structure shapes the evolution and epidemiology of *B. lusitaniae*, we analyzed the phylogeographic population structure of this tick-borne bacterium using a recently developed multilocus sequence typing (MLST) scheme based on chromosomal housekeeping genes. A total of 2,099 questing nymphal and adult *Ixodes ricinus* ticks were collected in two climatically different regions of Portugal, being ∼130 km apart. All ticks were screened for spirochetes by direct PCR. Attempts to isolate strains yielded 16 cultures of *B. lusitaniae* in total. Uncontaminated cultures as well as infected ticks were included in this study. The results using MLST show that the regional *B. lusitaniae* populations constitute genetically distinct populations. In contrast, no clear phylogeographic signals were detected in sequences of the commonly used molecular markers *ospA* and *ospC*. The pronounced population structure of *B. lusitaniae* over a short geographic distance as captured by MLST of the housekeeping genes suggests that the migration rates of *B. lusitaniae* are rather low, most likely because the distribution of mediterranean lizard populations is highly parapatric. The study underlines the importance of vertebrate hosts in the geographic spread of tick-borne microparasites.

## Introduction

Lyme borreliosis (LB) is a complex tick-borne zoonosis and the most frequent vector-borne disease of humans in the temperate zone of both the New and Old World. It is named after the town Old Lyme in coastal Connecticut, northeastern United States, where a cluster of cases of juvenile arthritis was observed in the 1970s. The agent was identified as a tick-borne spirochete of the genus *Borrelia* and named *B. burgdorferi* [1]. However, with the analysis of samples from other parts of the world, it soon became clear that LB spirochetes constitute a group of species, whose ecological and pathological properties vary substantially [2], [3].

The European species of the LB group of spirochetes display different patterns and levels of host specialization. For example, *B. valaisiana* and most *B. garinii* strains are maintained by birds, while *B. afzelii* is specialized to rodents [3], [4]. These host associations influence distribution and relative abundance of the spirochetal species [5] and are likely to shape the phylogeographic population structures within each species. It can be expected that *B. garinii* and *B. valaisiana* show pronounced spatial mixing due to high dispersal rates of migratory birds, whereas it is likely that *B. afzelii* displays intraspecific geographic structure due to low dispersal rates of rodents.

On the Iberian Peninsula several species of LB group spirochetes have been detected in *Ixodes ricinus* ticks, mainly *B. garinii*, *B. afzelii*, *B. valaisiana* and *B. lusitaniae* [6]–[9]. *B. garinii* and *B. afzelii* are known to be pathogenic in humans. *B. lusitaniae* has been shown to be pathogenic in laboratory mice [10] and has also been isolated from human patients [11].

While all these four species occur in central and northern parts of Portugal and Spain, *B. lusitaniae* is the sole species of the LB group in southern Portugal and North Africa [12]–[14]. Lizards of the family *Lacertidae* are now believed to be important reservoir hosts of *B. lusitaniae* [15], [16]. These reptiles are known to be highly structured phylogeographically, suggesting limited migration between populations from different localities [17]–[20]. This is likely to have implications for the evolution and epidemiology of *B. lusitaniae*.

LB group spirochetes have commonly been typed using single loci, such as different intergenic spacer regions (IGS) [2], [21], [22] or the genes encoding the outer surface proteins A (*ospA*) [23] and C (*ospC*) [24]. However, single-locus approaches have drawbacks in terms of inferring evolutionary relationships among the microbial populations [25], [26]. Multilocus sequence typing (MLST) [27] or multilocus sequence analysis (MLSA) (the latter refers to genus-wide analyses) [28] based on housekeeping genes are considered to be the most powerful genotyping tools in studies of the population biology of microbial organisms. In order to infer possible processes that shape the evolution and epidemiology of *B. lusitaniae* at a finer geographic scale in Portugal, we evaluated whether this bacterium is structured phylogeographically. For this, we applied a recently developed MLST scheme based on chromosomal housekeeping genes of *B. burgdorferi* [29] to samples of *B. lusitaniae* obtained from two regions of Portugal, Mafra and Grândola (Figure S1). In addition to MLST of the core genome, we analyzed the 5S–23S IGS, *ospA* and *ospC* of the *B. lusitaniae* samples. While phylogenetic analyses of *ospA* and *ospC* did not provide signals of geographic structuring of *B. lusitaniae*, the results obtained using MLST revealed that the *B. lusitaniae* populations from these two regions constitute genetically distinct subpopulations. This analysis, therefore, confirms the increased utility of multiple housekeeping genes for studies of the geographic population structure of LB group spirochetes and suggests an association between the population structure of the bacteria and that of their vertebrate hosts.

## Results

Based on sequence analyses of multiple housekeeping genes (i.e. *clpA*, *clpX*, *nifS*, *pepX*, *pyrG*, *recG* and *rplB*) of the *B. lusitaniae* samples analyzed in this study (Table 1), 13 sequence types (STs) were defined by MLST, and no ST was observed in more than two samples (Table 2). Among the housekeeping genes, the highest sequence diversity was noted in *clpA*, *pepX* and *rplB*, which also revealed high numbers of alleles (Table 3). The *nifS* gene was the least polymorphic of the housekeeping genes analyzed, with a percentage of variable sites of 1.06, the lowest number of alleles and also the lowest level of nucleotide diversity per site (Table 3). The average ratios of non-synonymous and synonymous substitutions (dN/dS) of the housekeeping genes and *ospA* were <1, indicating that they are nearly neutral or under purifying selection (Table 3). The MLST data have been submitted to the MLST website hosted at Imperial College London, United Kingdom (www.mlst.net), and can be accessed via strain ID or ST. For *ospC*, the dN/dS ratio was >1, suggesting that the gene encoding this outer surface protein is under some level of positive immune selection [24], [30].

**Table 1:** *B. lusitaniae* samples analyzed in this study.

Origin	Sample	Date of collection	Reference
Lisbon	PoHL1*	May 2002	[11]
Mafra	PoTiBL37*	April 1999	[9]
	PotiBmfP109#	May 2004	This study
	PotiBmfP220	April 2003	“
	PotiBmfJ2	January 2001	“
	PotiBmfJ50	January 2003	“
	PotiBmfP147	March 2003	“
	PotiBmfP364	December 2003	“
Grândola	PoTiBGr41*	November 2002	“
	PoTiBGr82* †	November 2002	“
	PoTiBGr128* #	February 2003	“
	PoTiBGr130*	February 2003	“
	PoTiBGr131*	February 2003	“
	PoTiBGr136*	February 2003	“
	PoTiBGr143*	February 2003	“
	PoTiBGr209*	March 2003	“
	PoTiBGr210#	March 2003	“
	PoTiBGr211*	March 2003	“
	PoTiBGr212#	March 2003	“
	PoTiBGr213*	March 2003	“
	PoTiBGr288*	April 2003	“
	PoTiBGr293*	April 2003	“
	PoTiBGr409*	May 2003	“

* *Borrelia* strains successfully cultured in BSKII medium.

# Samples excluded from the study due to multiple infections with different *Borrelia* strains.

† Sample excluded from the *ospC* analysis since no *ospC* sequence was obtained.

All the strains were detected in or isolated from *I. ricinus* ticks, except for the isolate PoHL1 that was obtained from a human skin biopsy.

**Table 2:** Allelic profiles and STs of *B. lusitaniae*.

*B. lusitaniae* samples	*clpA*	*clpX*	*nifS*	*pepX*	*pyrG*	*recG*	*rplB*	ST	IGS	*ospA*	*ospC*
PoTiBGr41	26	15	18	21	13	22	13	**60**	1	1	1
PoTiBGr82	27	16	18	22	13	23	14	**61**	2	2	ND
PoTiBGr130	27	17	18	22	13	23	14	**62**	2	2	2
PoTiBGr131	28	18	18	21	14	22	13	**63**	3	1	3
PoTiBGr136	29	19	18	23	14	24	15	**64**	4	1	4
PoTiBGr409	29	19	18	23	14	24	15	**64**	9	1	4
PoTiBGr143	30	15	18	21	13	23	16	**65**	5	1	5
PoTiBGr211	30	15	18	21	13	23	16	**65**	5	1	5
PoTiBGr209	26	20	18	22	15	22	17	**66**	6	3	6
PoTiBGr213	26	20	18	22	15	22	17	**66**	7	4	6
PoTiBGr288	28	15	18	24	16	22	18	**67**	8	5	7
PoTiBGr293	26	19	18	22	17	23	19	**68**	2	1	8
PoHL1	31	19	19	25	18	25	20	**69**	10	6	9
PoTiBL37	31	19	19	25	18	25	20	**69**	11	6	9
PotiBmfP147	31	19	19	25	18	25	20	**Lus1**	10	10	13
PotiBmfP220	32	18	18	22	14	26	14	**Lus2**	12	7	10
PotiBmfJ2	33	21	20	26	18	25	20	**Lus3**	10	8	11
PotiBmfJ50	33	21	20	26	18	25	20	**Lus3**	10	9	12
PotiBmfP364	34	22	21	27	19	25	21	**Lus4**	10	2	14

* STs 60–69 are based on eight housekeeping genes including *uvrA* and were defined according to the MLST website, www.mlst.net. Allele numbers of *uvrA* for STs 60–69 can be found in the website under strain ID. For the five samples where no *uvrA* data were available, alleles for the seven remaining housekeeping were also assigned allele numbers according to the website, however, STs were arbitrarily labelled Lus 1-4. Alleles of the IGS, *ospA* and *ospC* were assigned numbers in the order new alleles were found.

ND: not determined.

**Table 3:** Loci used for MLST and single locus typing.

Locus	Product	Primer sequence (5′–3′)	Amplicon size (bp)	MLST fragment size (bp)	G+C %	No. of alleles	dN/dS ratio	% VI	π
*clpA*	Clp protease subunit A	clpAF1240: GATAGATTTCTTCCAGACAAAG	864	579	25.7	9	0.308	2.94	0.01122
		clpAr2104: CAAAAAAAACATCAAATTTTVTATCTC							
*clpX*	Clp protease subunit X	clpXF403: AATGTGCCATTTGCAATAGC	721	624	30.7	8	0.026	1.92	0.00529
		clpXr1124: TTAAGAAGACCCTCTAAAATAG							
*nifS*	aminotransferase	nifSF1: ATGGATTTCAAACAAATAAAAAG	696	564	27.1	4	0.079	1.06	0.00446
		nifSr719: GTTGGAGCAAGCATTTTATG							
*pepX*	dipeptidyl aminopeptidase	pepXF449: TTATTCCAAACCTTGCAATCC	723	570	28.0	7	0.15	2.45	0.0080
		pepXr1172: GTTCCAATGTCAATAGTTTC							
*pyrG*	CTP synthase	pyrGF448: GATATGGAAAATATTTTATTTATTG	742	603	31.2	7	0	1.49	0.00549
		pyrGr1190: CAAACATTACGAGCAAATTC							
*recG2*	DNA recombinase	recGF917: CTTTAATTGAAGCTGGATATC	777	651	30.6	5	0.06	1.38	0.00530
		recGr1694: GAAAGTCCAAAACGCTCAG							
*rplB*	50S ribosomal protein L2	rplBF2: TGGGTATTAAGACTTATAAGC	758	624	36.1	9	0.085	2.40	0.00757
		rplBr760: GCTGTCCCCAAGGAGACA							
*ospA*	outer surface protein A	Fw1: GACACTGCCTCTGGTGATAGC	302	288	37.4	10	0.69	14.9	0.03050
		Rv1: CTTTCCCTTTTCCTTCTTTTG							
		Fw2: AAACAAAGACGGCAAATACGA							
		Rv2: ATTCAAGCTTGGTGCCATTT							
*ospC*	outer surface protein C	Fw1: TGAAAAAGAATACATTAAGTGCAA	470	376–385	35.7	14	2.24	36.6	0.14230
		Rv1: TTTTTGGAGTTTCTGCYACA							
		Fw2: TGGCTGTGAAAGAAGTTGAGG							
		Rv2: GCCACAACAGGACTTGTAAGC							
IGS 5S–23S	N/A	Reference 21	225	151–183	18.6	12	N/A	9.23	0.01806

N/A: not applicable.

**Figure 1:**
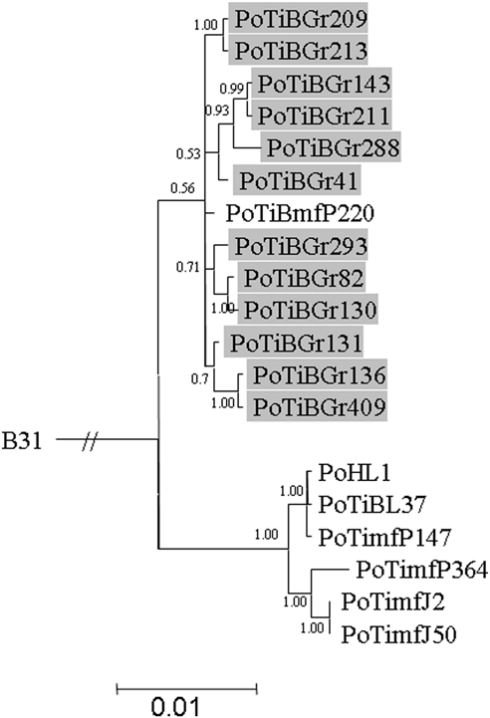
Bayesian phylogenetic tree of *B. lusitaniae* using concatenated sequences of *clpA*, *clpX*, *nifS*, *pepX*, *pyrG*, *recG*, *rplB*. The tree was rooted with *B. burgdorferi* strain B31. Posterior probabilities values are indicated to provide branch support. The scale bar represents 1% sequence divergence. *B. lusitaniae* samples derived from Grândola are highlighted.

The MLST tree generated in this study discriminates the Mafra samples of *B. lusitaniae* from the Grândola samples (Figure 1). The human isolate PoHL1 clusters together with samples from Mafra in 100% of the trees drawn from the posterior probability. Interestingly, PoTiBmfP220, a strain detected in Mafra, arises from the branch representing the Grândola samples (see below).

**Figure 2:**
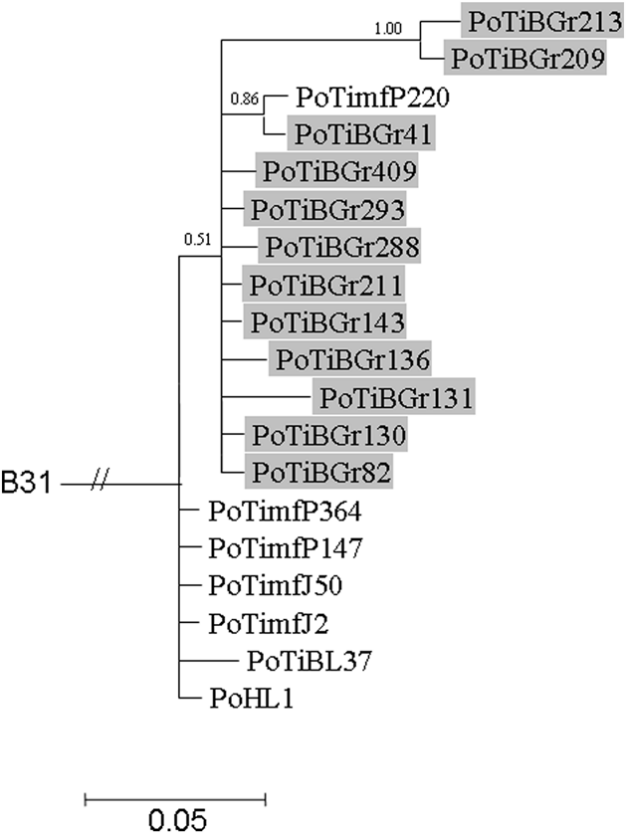
Bayesian phylogenetic tree of *B. lusitaniae* strains based on the IGS. The tree was rooted with *B. burgdorferi* strain B31. Posterior probabilities values are indicated to provide branch support. The scale bar represents 5% sequence divergence. *B. lusitaniae* samples derived from Grândola are highlighted.

**Figure 3:**
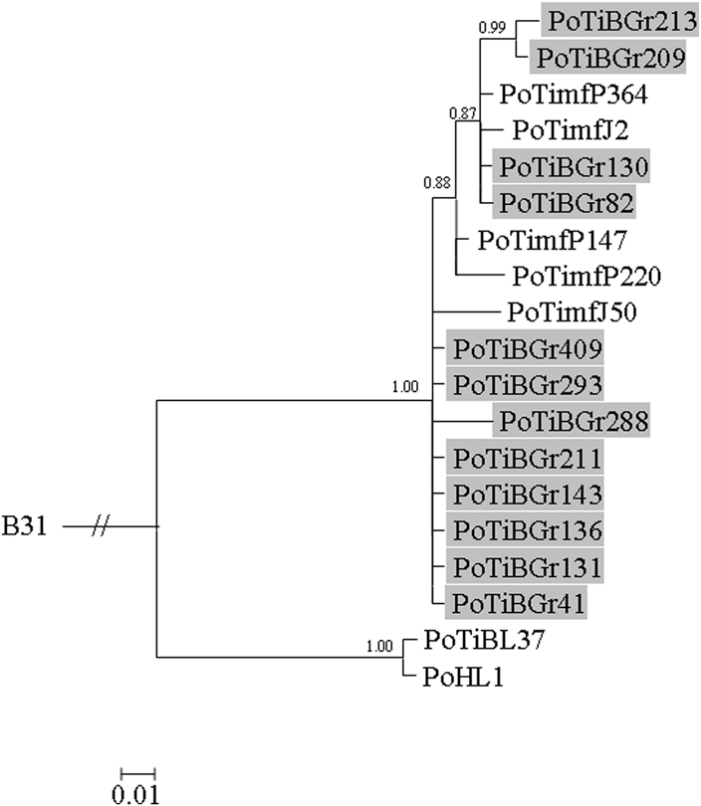
Bayesian phylogenetic tree of *B. lusitaniae* strains based on *ospA*. The tree was rooted with *B. burgdorferi* strain B31. Posterior probabilities values are indicated to provide branch support. The scale bar represents 1% sequence divergence. *B. lusitaniae* samples derived from Grândola are highlighted.

**Figure 4:**
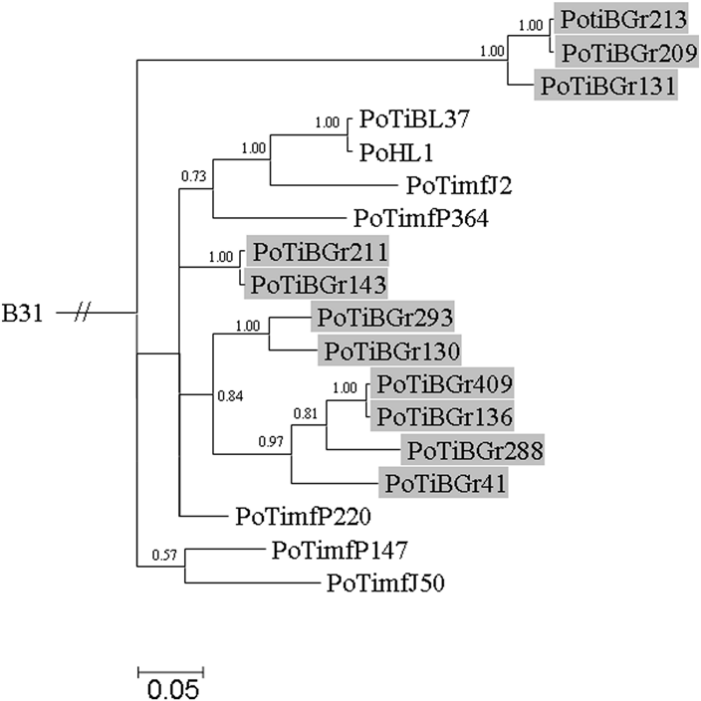
Bayesian phylogenetic tree of *B. lusitaniae* strains based on *ospC*. The tree was rooted with *B. burgdorferi* strain B31. Posterior probabilities values are indicated to provide branch support. The scale bar represents 5% sequence divergence. *B. lusitaniae* samples derived from Grândola are highlighted.

Signals of phylogeographic structuring were also found for the individual housekeeping genes and the IGS, but the intrapopulation phylogenies were less resolved (Figures S2, S3, S4, S5, S6, S7, S8, Figure 2). In contrast, the phylogenetic trees of *ospA* and *ospC* showed no clear signals of geographic structuring of the *B. lusitaniae* samples (Figures 3 and 4). For *ospC*, the lack of geographic structuring may be related to balancing selection and/or recombination. Consistent with this, apart from signatures of positive selection, several recombination events were detected in the *ospC* sequences using the RDP suite of programs. Recipient and donor strains, position in the alignment and P-values for the individual methods are shown in Table 4. Recombination events will influence the tree topology and may lead to the polytomies that are observed in the *ospC* tree (Figure 4). No recombination events were detected in *ospA* using RDP. (The sequences of the IGS, *ospA* and *ospC* have been deposited in the GenBank database under accession numbers EF179549 to EF179604.)

**Table 4:** Recombination at *ospC* of *B. lusitaniae* samples.

Recipient	Donor	Position	Gscale	Method	Av P-value
PoTimfJ2 (PotimfP147)*	PoTiGr130 (PotimfP147)*	40–178	0	GENECONV	–
				MaxChi	0.046
				Chimaera	0.0011
				SiScan	0.00062
				3SEQ	0.0054
				LARD	0.28
PoHL1/PoTiB37	PoTimfJ2	271–368	5	GENECONV	0.0039
				MaxChi	0.005
				Chimaera	0.01
				3SEQ	0.0022
				LARD	0.1

* In this analysis the status of strain PoTimfP147 was uncertain and designated as ‘minor parent’ (donor) or maybe ‘daughter’ (recipient).

**Figure 5:**
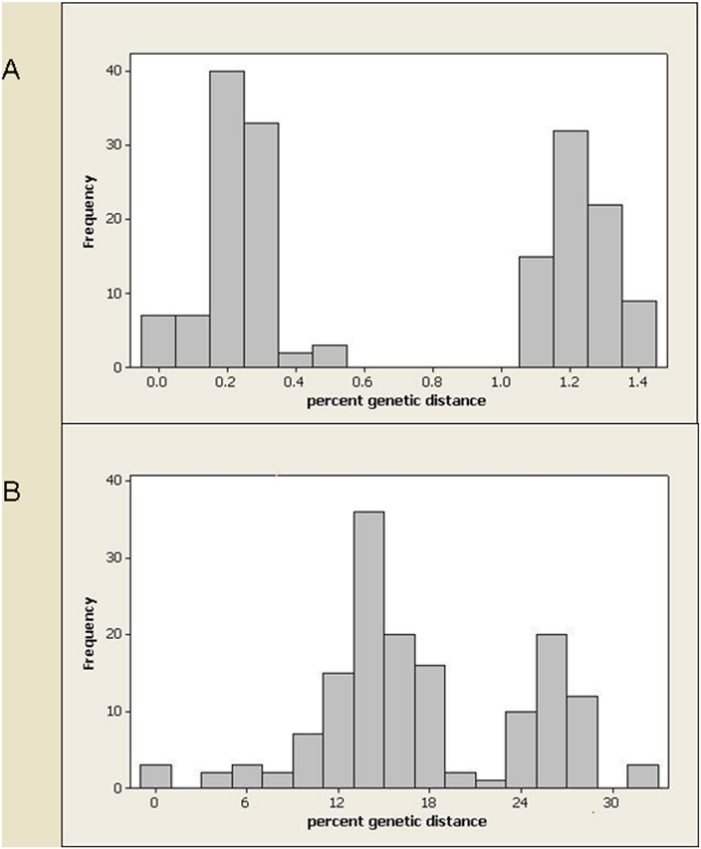
Distribution of pairwise genetic distance for *B. lusitaniae* housekeeping genes (A) and *ospC* (B).

An analysis of the pairwise divergences between the samples at the housekeeping genes and *ospC* also illustrates the difference between these loci. The distribution of pairwise differences of the concatenated housekeeping genes is bimodal (Figure 5A), with the peak at the lower distances representing intra-population comparisons and the peak at high distances representing inter-population comparisons. In contrast, although two peaks are still discernable in the distribution for *ospC*, these peaks are much less distinct (Figure 5B). When the same analysis was carried out for *ospA*, the distribution was notably bimodal (Figure S10A). However, this was predominantly due to the inclusion of two highly diverged strains at this locus, the human-derived strain PoHL1 and the tick isolate PoTiBL37, as the peak corresponding to large distances reflects comparisons involving one of these isolates. When these isolates were removed, the distribution was no longer bimodal (Figure S10B).

**Figure 6:**
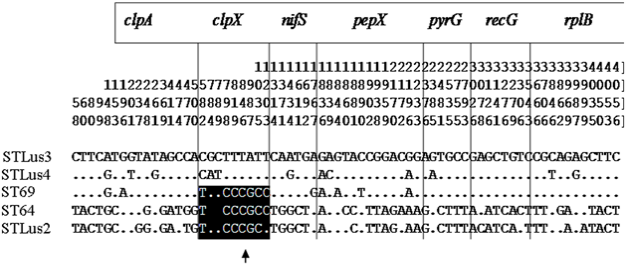
Variable sites for each housekeeping gene shown for ST64, ST69, STLus2, STLus3 and STLus4. The numbers above the sequences refer to the position in the concatenated alignment. Regions corresponding to the individual genes are separated by a line and gene names are given on the top. The likely recombination event between ST64 and ST69 in *clpX* is indicated by an arrow.

**Figure 7:**
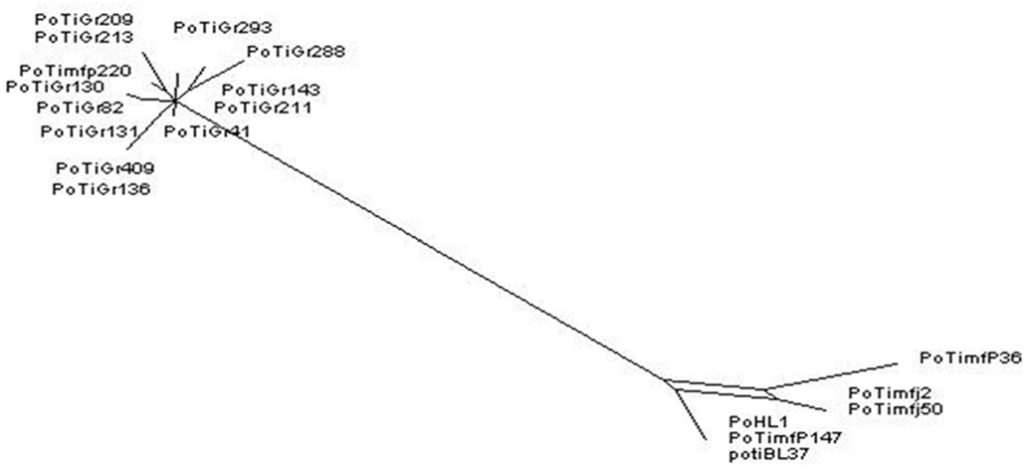
Network analysis. An analysis of *B. lusitaniae* MLST data (concatenated housekeeping gene sequences) using SplitDecomposition provided a network at the split separating the strains from Mafra which coincides with a recombination event in *clpX*. The two populations from Mafra and Grândola are well separated.

Although intragenic recombination was not detected in the individual housekeeping genes using the RDP suite of programs, a putative recombination event corresponding to the region of *clpX* was detected with RDP and Bootscan when the housekeeping gene sequences were concatenated (p = 0.019). Indeed, in the allelic profiles *clpX* allele 19 (Table 2) was found to be the only allele shared between ST69 from Mafra and ST64 and ST68 from Grândola (for alleles of strain PoTiBmf220, see below). The alignment of the polymorphic sites of the concatenated housekeeping genes demonstrated that *clpX* of ST64 (PoTiBGr136) and ST69 (e.g. PoHL1) had obviously recombined (Figure 6). To further investigate this, an analysis of the STs (as defined in Table 2) using ClonalFrame software confirmed a single recombination event for *clpX* on the branch above node D (Figures S11 and S12). At node D STLus3 and STLus4 are split, which correspond to samples PoTiBmfJ2, PoTiBmfJ50, and PoTiBmf364 (Table 2). A network analysis using the Splitstree software package produced an unresolved split separating the strains from Mafra which corresponds to the region above node D (Figure 7). Using the estimated value for θ of 23.46 in the ClonalFrame analysis, the inferred value of recombination to mutation, r/m, was estimated to be 0.01 with a 95% credibility region between 0.001–0.06. This suggests that recombination at the chromosome is very rare in *B. lusitaniae*.

Consistent with the pronounced phylogeographic signals captured in the MLST tree and the bimodal distribution of pairwise sequence distances, there was no intersection in the allelic profiles of the *B. lusitaniae* populations from the two regions, Mafra and Grândola, with the exception of the single recombination event mentioned above and of sample PoTiBmf220 (Table 2). This sample from Mafra shared five of the seven housekeeping genes with the Grândola samples. The remaining two housekeeping genes (i.e., *clpA* and *recG*) as well as the IGS, *ospA* and *ospC* of this strain were found to be unique.

In the dataset analyzed, only two STs (ST61 and ST62) were single locus variants, and all the others represented several diverse MLST profiles (Table 2). This finding indicates that the intraspecific diversity of *B. lusitaniae* is considerable, as already found in previous studies using the IGS [12], [14]. The genetic distances between samples from Mafra and Grândola, based on the housekeeping genes, were found to range from 0.0132 to 0.0137 (Table 5). This is lower than the genetic distance between the most divergent strains of *B. burgdorferi* [29], thereby supporting the rationale for considering the diverse *B. lusitaniae* populations analyzed in this study as conspecific.

**Table 5:** Pairwise genetic distance among of housekeeping genes of selected *B. lusitaniae* samples and *B. burgdorferi* strains B31, NE49 and Z41293.

Strain	B31	NE49	Z41293	PoTiBL37	PoTimfP364	PoTimfP220
B31		0.024*	0.021*			
NE49	0.0166(0.0183)					
Z41293	0.0152(0.0170)					
PoTiBGr41	0.0780			0.0137	0.0132	0.0024
PoTiBL37	0.0798				0.0048	0.0113
PoTimfP364	0.0800					0.0132

* pairwise genetic distance for multiple genes as determined by Postic and colleagues [52].

The values in brackets are based on eight housekeeping genes, including *uvrA*. The pairwise genetic distance among samples from Mafra ranged from 0.0002–0.0036, except for PoTimfP220, whereas these values ranged from 0.0–0.0048 for samples from Grândola.

## Discussion

MLST and MLSA are the most powerful tools for analyzing the evolution and population biology of microbial populations [28], [31]. Most MLST/MLSA schemes used so far have been applied to directly transmitted pathogens. Because the majority of indirectly transmitted zoonotic microparasites are maintained by wildlife and vectors, such as ticks or mosquitoes, environmental factors are particularly important in shaping their evolution and spread. As this may result in geographic structuring, genotyping methods of vector-borne microbial organisms should have the power to capture phylogeographic structure and to infer species trees. Using a novel MLST scheme, we have recently demonstrated that North American and European populations of LB group spirochetes are genetically distinct [29]. We have, furthermore, provided evidence that *B. burgdorferi* originated in Europe and not in North America [29]. Here we analyzed *B. lusitaniae* samples from two climatically different regions of Portugal (Figure S1) using this MLST scheme, suggesting that the two regional *B. lusitaniae* populations represent genetically distinct lineages. In contrast, no robust phylogeographic signals were observed for *ospA* and *ospC*.

The numbers of *B. lusitaniae* strains that were successfully cultured differed remarkably between the two regions, despite the fact that isolation attempts were made by the same person and method. While the bacterial populations may have subtle differences in metabolic requirements, previous studies have demonstrated that the infection prevalence of *B. lusitaniae* in ticks from Mafra is orders of magnitudes lower than in those from Grândola [9], [12]. This may explain the disparity in the number of isolates obtained. We, therefore, included infected ticks, from which the spirochetal genes of interest were amplified directly without prior culturing.

Although both Network analysis and allelic admixture analysis (ClonalFrame) indicated recombination events at one housekeeping gene (*clpX*), the overall ratio of recombination to mutation was very low, suggesting that the linear chromosome of *B. lusitaniae* is relatively clonal.

In another study the heterogeneity of *B. lusitaniae* was examined at a broader geographic scale compared with our study using *ospA* as marker [32], suggesting the existence of two major lineages in the Mediterranean Basin. According to that study, Italian and German strains form a ‘European’ lineage and the Portuguese strains PotiB1-3 and North African strains an ‘African’ lineage, with the Portuguese patient isolate PoHL1 being an exception as it was placed in the European clade. However, our findings indicate that the commonly used molecular marker *ospA* is not suitable for phylogeographic analyses of *B. lusitaniae* at a smaller geographic scale (Figure 3, Figure S9). The reasons for the lack of clear geographic signals contained in the *ospA* sequences remain unknown, since no recombination events were detected for this gene.

*ospC* is another popular molecular marker of LB group spirochetes [24], [30], [33]. As for *ospA*, however, analyses of *ospC* did not reveal signals of geographic structure of *B. lusitaniae* at a small geographic scale. Recombination and balancing selection are possible processes that homogenize the spatial frequency distribution of *ospC* alleles of *B. lusitaniae*, but either of these processes may generate a uniform geographic structure. As recombination has been detected and the dN/dS ratios of *ospC* were >1, we hypothesize that both processes shape the population structure of this gene.

The lack of geographic structuring observed at *ospA* and *ospC* may allow to draw the conclusion that the two *B. lustaniae* populations analyzed in this study are spatially mixed and that this bacterial species is not structured phylogeographically at the scale analyzed. On the other hand, the fact that the two populations from Mafra and Grândola do not share STs strongly indicates that the two populations presently do not, or very rarely, migrate between the two regions. It is possible that the patterns seen at the outer surface protein genes reflect ancient events that arose in a continuously distributed ancestral population (discussed below).

Given that bacterial housekeeping genes typically evolve very slowly (rates of synonymous substitution per site and year ∼10^−8^), it is likely that the bacterial populations have been separated from each other for a long time. It is possible that the two geographic clades of *B. lusitaniae* as demarcated by MLST represent diverged descendants of a common ancestral population prevailing during past glacial maxima. Given that Mafra and Grândola are only ∼130 km apart, isolation of these *B. lusitaniae* populations by distance alone is an unlikely explanation for the observed genetic divergence. It is plausible to assume that these local populations diverged through vicariance, because climate change after the last Ice Ages has generated ecological barriers between Mafra and Grândola. There is palaeobotanic evidence that during the last glacial maximum most of Portugal was covered by temperate mixed forests [34], whereas the present day climate and vegetation of southern Portugal, but not that of Mafra, resembles that of the African Maghreb [9]. Postglacial ecological differences between Mafra and Grândola, including those imposed by more recent human activities, are likely to have shaped the population structure and biogeographic patterns of vertebrate host communities, in particular reptilian populations. Furthermore, the river Tejo is likely to act as firm present-day barrier to migration of terrestrial reptiles between Mafra and Grândola (Figure S1). A number of studies have, in fact, revealed that the reptilian populations in the Mediterranean Basin are highly structured genetically and that their distribution is parapatric [17]–[20]. Because lizards are now considered important (if not the exclusive) reservoir hosts of *B. lusitaniae* [14]–[16], their limited dispersal will affect the migration rates of *B. lusitaniae*, resulting in the observed fine-scale geographic structure of this tick-borne bacterium. Although *I. ricinus* ticks infected with *B. lusitaniae* may be dispersed rapidly over long distances when feeding on highly mobile hosts, such as migratory birds, this is unlikely to be an important process in the effective dispersal of *B. lusitaniae*. Feeding tick larvae apparently do not acquire *B. lusitaniae* from vertebrate species other than lizards. On the other hand, *B. lusitaniae*-infected nymphs that feed on long-distance migrants will give rise to questing adult ticks that subsequently feed on larger animals, such as deer, which are not reservoir competent for any of the species of the LB group of spirochetes [3], [4]. Thus, only larvae and nymphs that feed on lizards will maintain the cycles of *B. lusitaniae*. In other words, the migration rates of *B. lusitaniae* are determined by those of lizards.

In central and northern parts of the Iberian Peninsula *B. garinii*, *B. valaisiana* and *B. afzelii* have been recorded in addition to *B. lusitaniae* [7]–[9], a pattern of species richness that is similar to that recorded for Central Europe [3], [35]–[37]. The presence of these species strongly suggests that rodents and birds are also involved in the ecology of LB in central and northern Portugal [4]. In contrast, *B. lusitaniae* is the only species of the LB group in southern Portugal and North Africa [12]–[14]. It is interesting to note that the infection prevalence of *B. lusitaniae* in southern Portugal and North Africa was found to be much higher than the overall infection prevalence of all species of the LB group taken together in other parts of the world, including the Mafra region of Portugal [3], [9], [12]–[14], [37]. This might indicate the operation of the ‘dilution effect’ in central and northern Portugal due to a more diverse vertebrate community which *I. ricinus* ticks parasitize [38]. The apparent lack of *B. garinii*, *B. valaisiana* and *B. afzelii*, but high infection prevalence of *B. lusitaniae*, in southern Portugal and North Africa suggests that immature *I. ricinus* ticks in that region feed mainly on reptilian hosts, allowing for considerable amplification of *B. lusitaniae*.

Taken together, the study strongly supports the idea that levels and patterns of host specialization of vector-borne microparasites affect their emergence and geographic spread. Population and landscape genetic studies of other vector-borne systems are needed to test the generality of this idea.

## Materials and Methods

### Tick collection and habitat description

Questing *I. ricinus* ticks were collected between 2001 and 2004 by blanket dragging in sylvatic habitats in Mafra (Estremadura region, ∼25 km north of Lisbon, Portugal; 1,598 nymphal ticks, 413 adult ticks) and in Grândola (Alentejo region, ∼100 km south of Lisbon; 88 adult ticks (40 male, 48 female ticks) (Figure S1). The climate in Mafra is temperate and humid, influenced by the Atlantic. The dense woodland habitats consist of deciduous oaks (*Quercus faginea*), eucalyptus, pine and chestnuts with well developed herbage layers. The Mafra site was located inside a park that was created in the 18^th^ century and served as leisure and hunting site for the Portuguese royalty. Movements of large animals into and out of the park are restricted. The climate in Grândola is more mediterranean and drier than that in Mafra. Cork oaks (*Q. suber*) are common [9].

### Screening for spirochetes and culturing

After decontamination, the ticks were cut into two halves under aseptic conditions. One half was inoculated into BSK-II media to obtain isolates. The remaining halves of the ticks were analyzed for infection by direct PCR targeting the 5S–23S IGS of the spirochetes [21]. The samples were assigned to *Borrelia* species using restriction fragment length polymorphism as described previously [2]. One *B. lusitaniae* culture was obtained from the 2,011 ticks collected in Mafra, and 15 *B. lusitaniae* cultures from the 88 adult ticks collected in Grândola. While in Grândola *B. lusitaniae* is relatively abundant and the sole LB species found [12], its prevalence in Mafra is very rare which is reflected in the limited number of isolates obtained from this region [9]. Only uncontaminated cultures and a subset of ticks found to be infected with *B. lusitaniae* were included in this study (Table 1). Isolation of LB spirochetes is carried out in liquid media, and cloning procedures using subsurface plating on solid media are difficult to perform and not carried out routinely. Mixed infections were, therefore, excluded at the stage of sequence analysis (see below).

### PCR and sequencing

MLST was performed on cultured isolates of *B. lusistaniae* and directly on some infections in ticks without prior isolation of the spirochetes. The original MLST scheme developed for *B. burgdorferi* by Margos and colleagues [29] comprised eight housekeeping genes, i.e. *clpA*, *clpX*, *nifS*, *pepX*, *pyrG*, *recG*, *rplB* and *uvrA*. For five tick-derived *B. lusitaniae* samples, *uvrA* could not be amplified using a single pair of PCR primers and, therefore, most analyses in this study were carried out without *uvrA*. In addition, *ospA*, *ospC* and the 5S-23 IGS were amplified and sequenced. The PCR primers used in this study are shown in Table 3.

For DNA preparation, cultured *Borrelia* strains (1×10^7^ spirochetes) were centrifuged at 13,000 rpm for 20 min, resuspended in 1 ml of PBS buffer and heated to 100°C for 10 min. For PCR amplification of the housekeeping genes, a 1/1000 dilution of these preparations was used as DNA template. PCR reactions were performed in 50 µl volume of 1× reaction mix (BioMix Red, BIOLINE, United Kingdom), 25 pmol of each primer and 2.5 µl of template DNA. The amplification conditions were as follows: 2 min of initial denaturation at 95°C, then 40 cycles at 95°C for 30 s, 50°C for 30 s, and 72°C for 1 min. The amplification was completed by a final step of 5 min at 72°C to allow complete extension of all PCR products.

PCR amplification of the housekeeping genes from tick lysates as templates were performed using HotstarTaq Mastermix (Qiagen, Germany) under the following conditions: initial denaturation at 95°C for 15 min, 10 cycles of 95°C for 30 s, 52°C for 30 s, 72°C for 1 min, and then 30 cycles of 95°C for 30 s, 50°C for 30 s, 72°C for 1 min, and a final elongation step of 72°C for 5 min.

In parallel with MLST of the core genome, the IGS, *ospA* and *ospC* were analyzed. To amplify the latter two genes, two sets of primers were designed for a nested PCR approach (Table 3). Amplification of the IGS and the *osps* was carried out in a 50 µl reaction mixture containing 1 pmol of each primer, 200 µM (each) dATP, dGTP, dCTP and dTTP (Invitrogen, United States), 1.75 U of Taq polymerase (Invitrogen, United States), 2 mM MgCl_2_, 0.5× BSA, 1× Taq buffer. To amplify the IGS, we used the primers and the PCR conditions described elsewhere [21]. For *ospA* and *ospC*, the first round of amplification was carried out using a touchdown protocol; after an initial denaturation step of 95°C for 5 min, 2 cycles of 95°C for 1 min, 64°C for 1 min, 72°C for 1 min were run, followed by decreasing the annealing temperature by 1°C per 2 cycles until reaching an annealing temperature of 55°C, used for the next 17 cycles. The final extension was set at 72°C for 5 min. A dilution of 1/100 of the first PCR product was used for the second set of PCR cycles. An initial cycle of denaturation for 5 min at 95°C was followed by 35 cycles of 95°C for 1 min, 50°C for 1 min and 72°C for 1 min and a final elongation step at 72°C for 5 min.

The amplified products were purified and sequenced. The DNA sequences were analyzed using the software package DNASTAR Lasergene 7 (DNASTAR Inc., United States). Samples providing ambiguous sequences were re-amplified and/or re-sequenced. Mixed infections in samples were readily revealed by analyses of the electropherograms of the housekeeping genes and excluded from this study.

For some strains, sequencing of *ospA* and/or *ospC* directly from PCR products was difficult. Therefore, these PCR products were cloned into a T-vector (pGEM-T, Promega, United Kingdom). Thereafter, several clones were sequenced using the universal T7 and SP6 primers. For the strain PoTiBGr82, we could not clone the *ospC* fragment, thus, no sequence data of this locus is available for this isolate.

### Analysis of MLST data

G+C content, percentage of variable sites (VI) and average number of nucleotide differences per site (π) were calculated for each locus using DnaSP version 4.0 [39]. Average dN/dS ratios were estimated using the modified Nei-Gojobori/Jukes-Cantor method in MEGA 4 [40]. MEGA 4 was also used to determine pairwise genetic distances. This approach was used to help calibrate the threshold levels of sequence divergence used to delineate species. In addition, the distance matrices based on the concatenated housekeeping gene, *ospC* and *ospA* sequences obtained with MEGA were transferred into Minitab Statistical Software® (Minitab Inc., State College PA, U.S.A.) to generate histograms of the frequency of genetic distances in *B. lusitaniae*.

Sequences of the housekeeping genes were assigned allele numbers. For those samples for which eight housekeeping genes could be amplified and sequenced, STs were defined according to the MLST website hosted at Imperial College London, United Kingdom (www.mlst.net). For the five samples for which no *uvrA* sequence information was obtained, sequences of the remaining seven genes were also assigned allele numbers according to the website, but STs were arbitrarily labelled as Lus1-4, because the *Borrelia* MLST website can only define STs if eight housekeeping genes are available.

Phylogenies were inferred for the concatenated sequences of the housekeeping genes and, individually, for the IGS, *ospA* and *ospC*. All alignments were made using MEGA 4. Phylogenetic trees were constructed using MrBayes software version 3.0b4 [41]. Sequences of the North American *B. burgdorferi* strain B31 were used to root the trees. Hierarchical likelihood ratio tests were conducted using MrModeltest (http://www.abc.se/~nylander) to provide the evolutionary models used in the Bayesian analysis. The models selected were GTR for r*ecG* and IGS, GTR+I for *clpX*, *pepX* and *ospA*, GTR+G for *clpA*, *pyrG* and *ospC*, and HKY+I for *nifS* and *rplB*. For the analysis of the concatenated sequences of the housekeeping genes, the GTR+G+I model was used. Each MrBayes analysis consisted of 2×10^6^ generations or until the standard deviation of split frequencies was <0.01 from a random starting tree and four Markov chains (with default heating values) sampled every 500 generations. To prevent reaching only apparent stationarity, two separate runs were made for each analysis. The first 1,000 sampled trees were discarded, resulting in a set of 3,000 analyzed trees sampled after stationarity.

### Detection of recombination in sequence data of *B. lusitaniae*

Sequences of *B. lusitaniae* (housekeeping genes, *ospA* and *ospC*) were tested for putative recombination events using Recombination Detection Program, version 3 (RDP3) [42]. The housekeeping genes were tested individually and as concatenated sequences.

In the RDP suite of programs a number of different methods are implemented and can be used simultaneously. The methods chosen for recombination detection in *B. lusitaniae* sequences included RDP [42], GENECONV [43], Maximum Chi Square (MaxChi)[44], [45], Chimaera [45], Sister Scanning (SiScan) [46], and 3SEQ [47] which constitute the most powerful methods currently available. Likelihood Assisted Recombination Detection (LARD) [48] was used to confirm potential recombination events detected by other methods.

To test *B. lusitaniae* sequences, the general settings were as follows: the highest acceptable P-value was set to 0.05 with Bonferroni corrections. In RDP the window size was set to 30 and the setting ‘internal and external references’ was chosen as recommended for small datasets (RDP3 Instruction Manual). In MaxChi the ‘variable site per window’ was set to 70, and ‘strip gaps’ switched on. In Chimaera the ‘variable sites per window’ was set to 60; and in SiScan the window size was set to 150 with a step size of 40. Two different analyses were done with identical setting for these programs. For GENECONV, one analysis was done with GSCALE set to 0, while in the second analysis GSCALE was set to 5 (which apparently is better to detect more ancient recombination events). Recombination events that were detected by more than two methods were confirmed with LARD, and P-values are given in Table 4.

ClonalFrame is a model-based method which was developed specifically for the analysis of multilocus sequence typing data to infer the clonal relationship of bacteria. The method allows to infer the chromosomal position of recombination events, to estimate the degree of relatedness of bacterial strains at different timescales and to reveal information on when strains last shared a common ancestor [49], [50]. To run ClonalFrame, an input file was created containing the sequences of STs for each individual housekeeping gene. Because ClonalFrame cannot estimate the value for θ, Watterson's θ (per sequence) was determined in DnaSP [39] using the concatenated housekeeping gene sequences. The concatenated housekeeping gene sequences were used in the Splitstree software package [51] to perform a network analysis using SplitDecomposition.

## Supporting Information

Figure S1 Map of Portugal showing the sampling sites Mafra and Grândola.
(0.08 MB PPT)

Figure S2 Bayesian phylogenetic inference for clpA of B. lusitaniae.
(0.06 MB PPT)

Figure S3 Bayesian phylogenetic inference for clpx of B. lusitaniae.
(0.06 MB PPT)

Figure S4 Bayesian phylogenetic inference for nifS of B. lusitaniae.
(0.06 MB PPT)

Figure S5 Bayesian phylogenetic inference for pepX of B. lusitaniae.
(0.06 MB PPT)

Figure S6 Bayesian phylogenetic inference for pyrG of B. lusitaniae.
(0.06 MB PPT)

Figure S7 Bayesian phylogenetic inference for recG of B. lusitaniae.
(0.06 MB PPT)

Figure S8 Bayesian phylogenetic inferences for rplB of B. lusitaniae.
(0.06 MB PPT)

Figure S9 Bayesian phylogenetic inference for ospA of B. lusitaniae, including samples from Italy and the Portuguese strains Poti B1-3. The figure shows that the Portuguese human isolate PoHL1 clusters with Italian samples (ITAh01, ITAh02; ‘European’ lineage), whereas samples from Mafra and Grândola cluster together with strains PotiB1-3 (‘African’ lineage (32)). Using MLST the Portuguese human isolate PoHL1 clusters with samples from Mafra.
(0.07 MB PPT)

Figure S10 Distribution of pairwise genetic differences at ospA of B. lusitaniae. The distribution of all samples included shows a bimodal distribution (A). Upon removal of strains PoHL1 and PoTiBL37, this distribution was not bimodal anymore, indicating that ospA does not clearly separate the regional B. lusitaniae populations (B).
(0.10 MB PPT)

Figure S11 Analysis of MLST sequences with ClonalFrame software. The figure shows the inferred genealogy of STs. The numbers of STs correspond to numbers as shown in Table 2. The nodes are labelled with letters A to L.
(0.08 MB PPT)

Figure S12 Probability plots for recombination. Each diagram corresponds to likely substitution/recombination events inferred on the branches above nodes C to L in Figure S11 (e.g. events on the branch above node C). For nodes A and B no diagram was obtained. The columns in each diagram correspond to each of the seven housekeeping gene fragments. The scale on the y axis ranging from 0 to 1 refers to the probability of recombination. The height of the red lines represents the inferred probability for recombination. Only for clpX on the branch above node D a recombination event was inferred. The black crosses indicate inferred substitutions, their intensity being proportional to their probability.
(0.25 MB PPT)
