# Supplementary figures and images for: Fine-Scale Phylogeographic Structure of Borrelia lusitaniae Revealed by Multilocus Sequence Typing
Source: PLoS One. 2008 Dec 23;3(12):e4002. doi: 10.1371/journal.pone.0004002 (PMC2602731; doi:10.1371/journal.pone.0004002)

## Slide 1
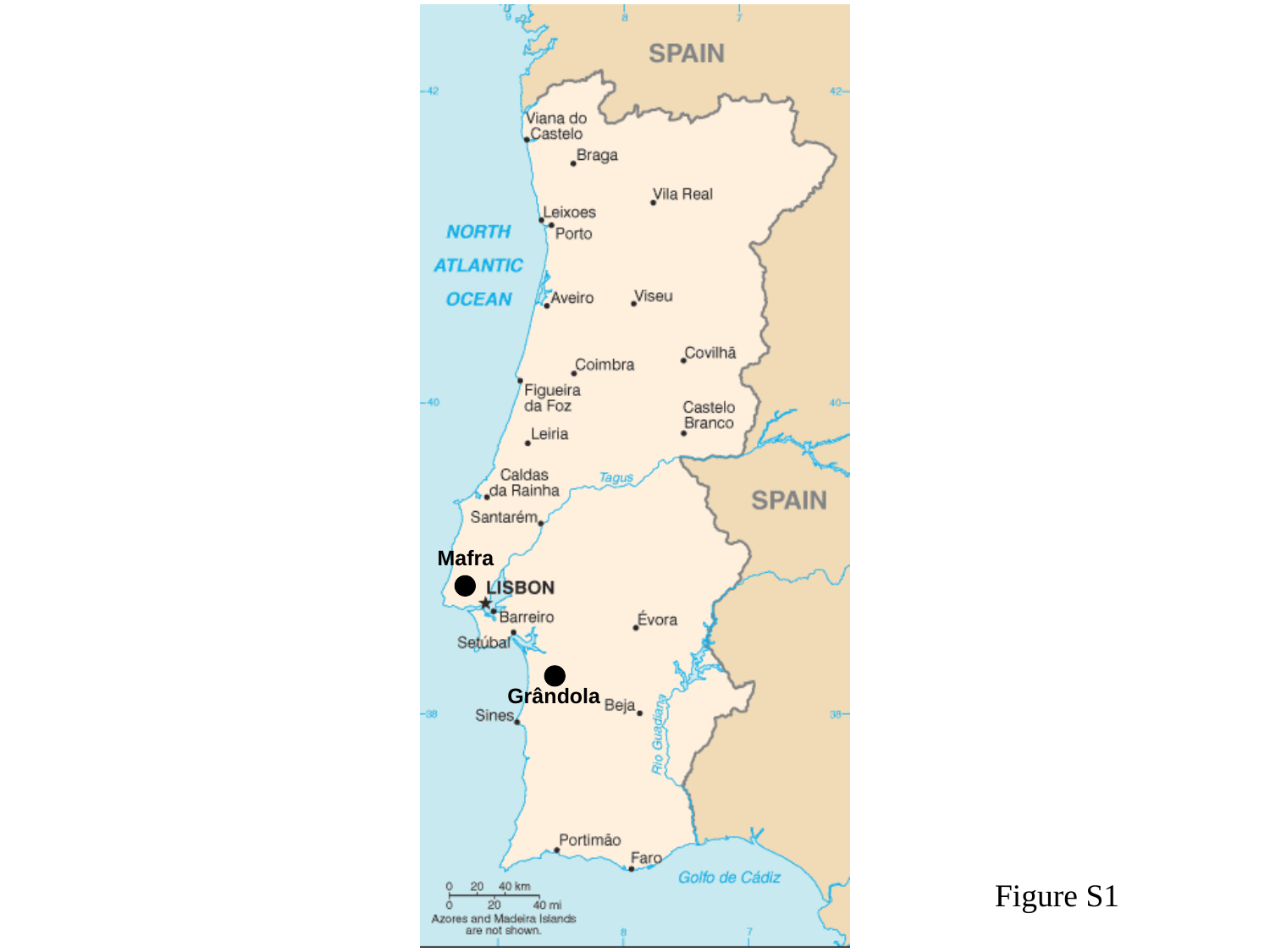

Mafra
Grândola
Figure S1

Supplement: Figure S1 — Map of Portugal showing the sampling sites Mafra and Grândola. (0.08 MB PPT) [file pone.0004002.s001.ppt]

## Slide 1
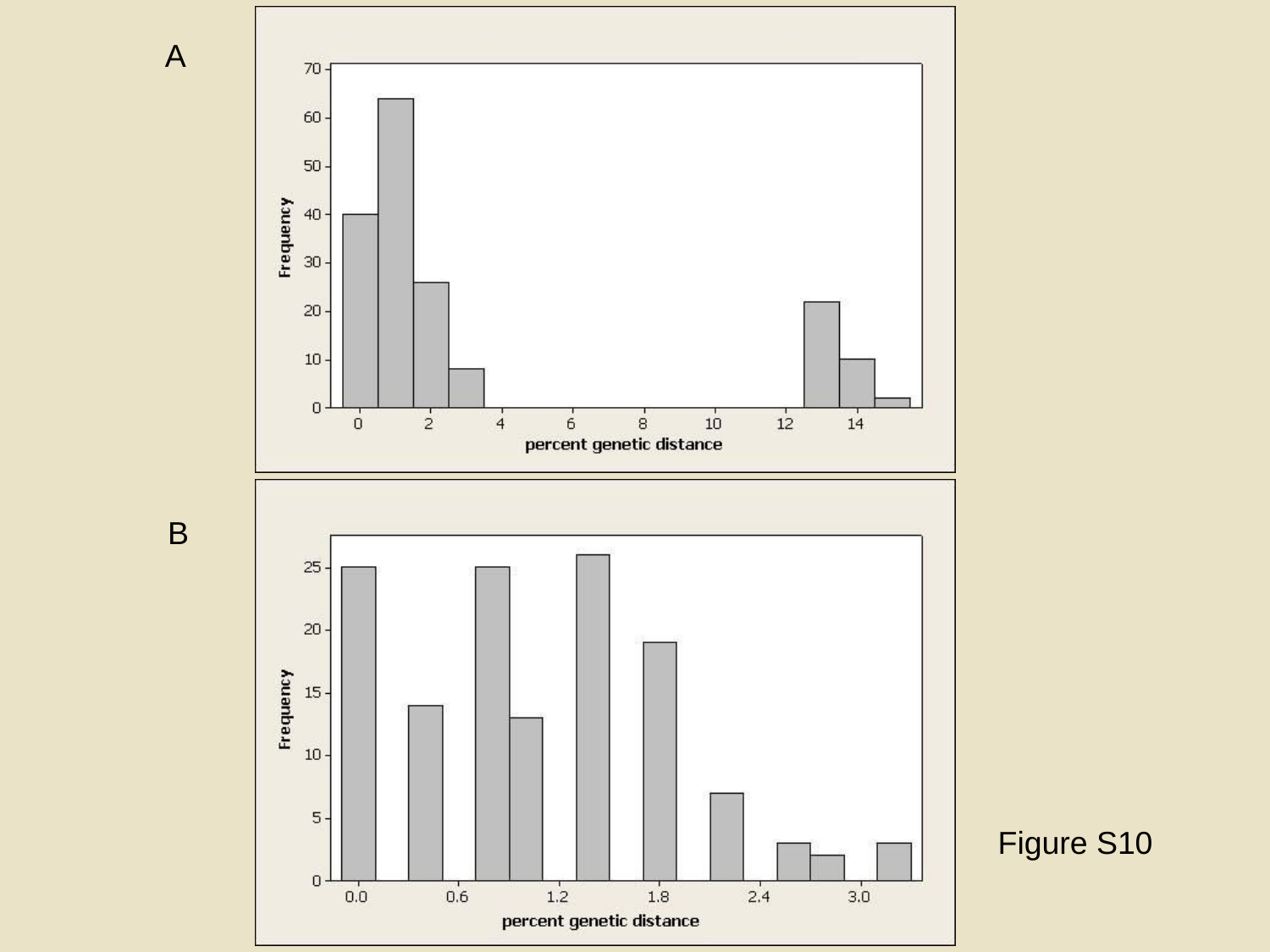

A
B
Figure S10

Supplement: Figure S10 — Distribution of pairwise genetic differences at ospA of B. lusitaniae. The distribution of all samples included shows a bimodal distribution (A). Upon removal of strains PoHL1 and PoTiBL37, this distribution was not bimodal anymore, indicating that ospA does not clearly separate the regional B. lusitaniae populations (B). (0.10 MB PPT) [file pone.0004002.s010.ppt]

## Slide 1
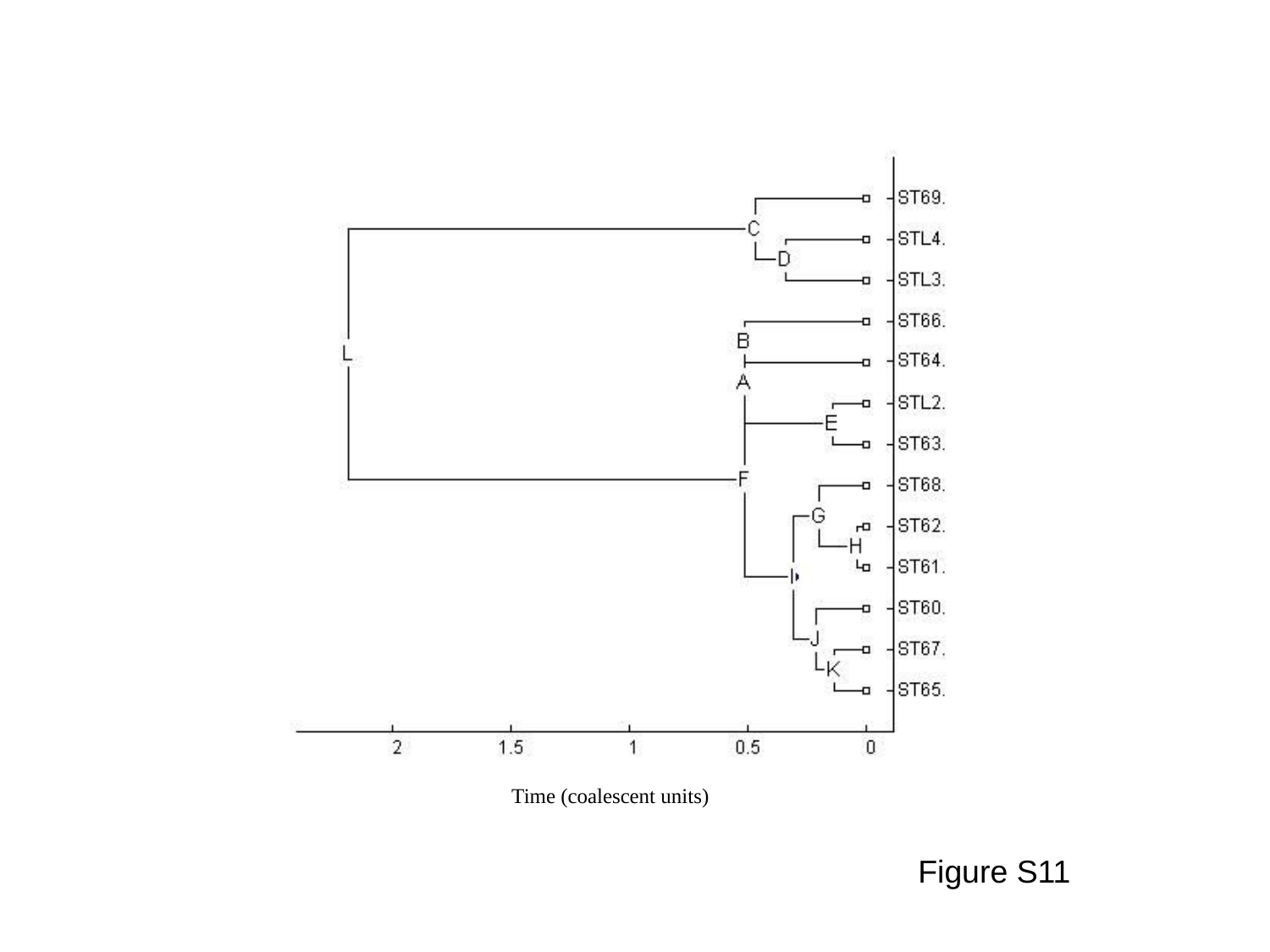

Time (coalescent units)
Figure S11

Supplement: Figure S11 — Analysis of MLST sequences with ClonalFrame software. The figure shows the inferred genealogy of STs. The numbers of STs correspond to numbers as shown in Table 2. The nodes are labelled with letters A to L. (0.08 MB PPT) [file pone.0004002.s011.ppt]
