# Supplementary material for: Fine-Scale Phylogeographic Structure of Borrelia lusitaniae Revealed by Multilocus Sequence Typing
Source: PLoS One. 2008 Dec 23;3(12):e4002. doi: 10.1371/journal.pone.0004002 (PMC2602731; doi:10.1371/journal.pone.0004002)
Supplement: Figure S3 — Bayesian phylogenetic inference for clpx of B. lusitaniae. (0.06 MB PPT) [file pone.0004002.s003.ppt]

## Slide 1
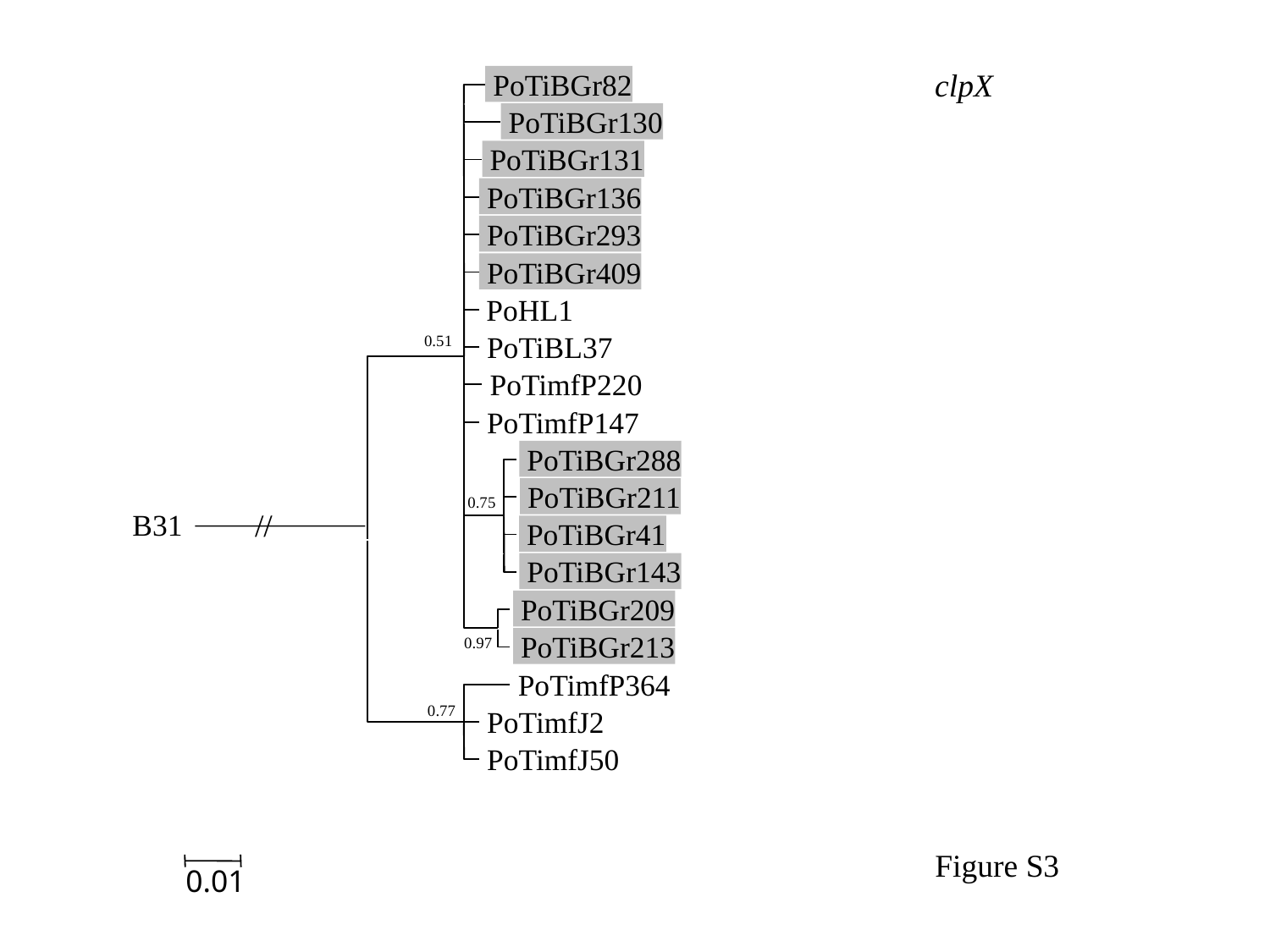

clpX
 PoTiBGr82
 PoTiBGr130
 PoTiBGr131
 PoTiBGr136
 PoTiBGr293
 PoTiBGr409
 PoHL1
 PoTiBL37
 PoTimfP220
 PoTimfP147
 PoTiBGr288
 PoTiBGr211
 PoTiBGr41
 PoTiBGr143
 PoTiBGr209
 PoTiBGr213
 PoTimfP364
 PoTimfJ2
 PoTimfJ50
0.51
0.75
//
 B31
0.97
0.77
Figure S3
0.01
