# Supplementary material for: Fine-Scale Phylogeographic Structure of Borrelia lusitaniae Revealed by Multilocus Sequence Typing
Source: PLoS One. 2008 Dec 23;3(12):e4002. doi: 10.1371/journal.pone.0004002 (PMC2602731; doi:10.1371/journal.pone.0004002)
Supplement: Figure S5 — Bayesian phylogenetic inference for pepX of B. lusitaniae. (0.06 MB PPT) [file pone.0004002.s005.ppt]

## Slide 1
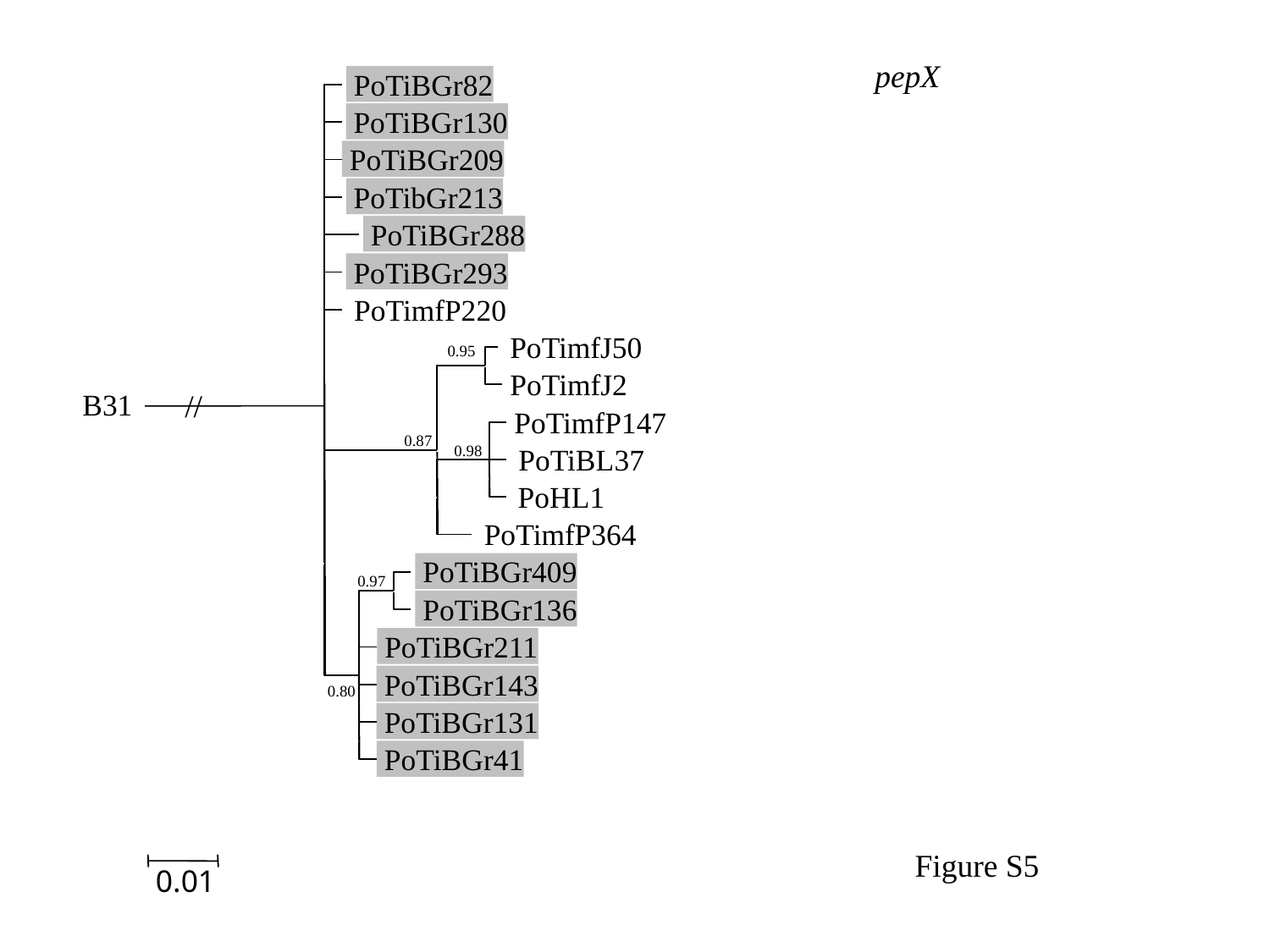

pepX
 PoTiBGr82
 PoTiBGr130
 PoTiBGr209
 PoTibGr213
 PoTiBGr288
 PoTiBGr293
 PoTimfP220
 PoTimfJ50
 PoTimfJ2
 PoTimfP147
 PoTiBL37
 PoHL1
 PoTimfP364
 PoTiBGr409
 PoTiBGr136
 PoTiBGr211
 PoTiBGr143
 PoTiBGr131
 PoTiBGr41
0.95
//
 B31
0.87
0.98
0.97
0.80
Figure S5
0.01
