# Supplementary material for: Fine-Scale Phylogeographic Structure of Borrelia lusitaniae Revealed by Multilocus Sequence Typing
Source: PLoS One. 2008 Dec 23;3(12):e4002. doi: 10.1371/journal.pone.0004002 (PMC2602731; doi:10.1371/journal.pone.0004002)
Supplement: Figure S8 — Bayesian phylogenetic inferences for rplB of B. lusitaniae. (0.06 MB PPT) [file pone.0004002.s008.ppt]

## Slide 1
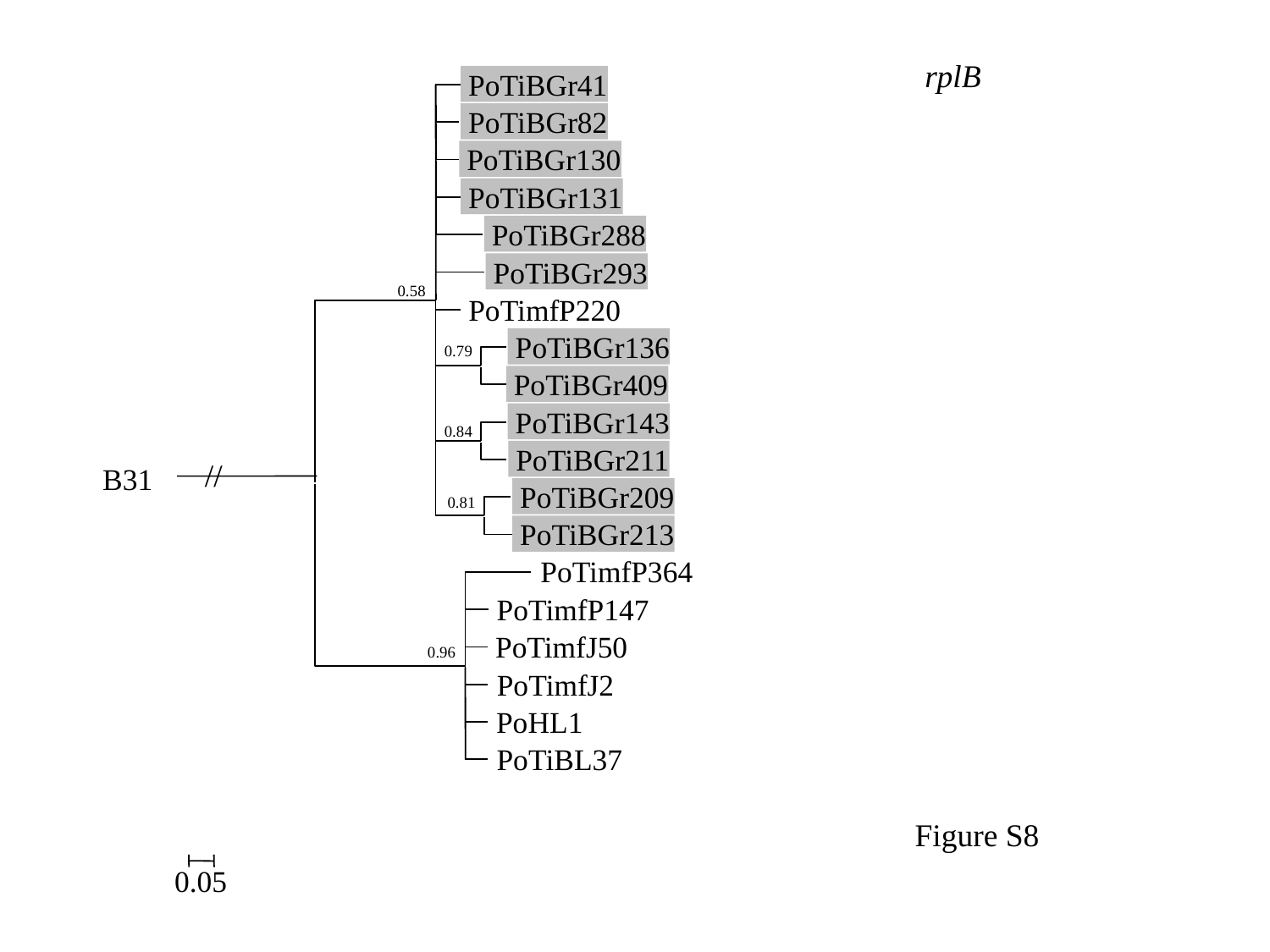

rplB
 PoTiBGr41
 PoTiBGr82
 PoTiBGr130
 PoTiBGr131
 PoTiBGr288
 PoTiBGr293
 PoTimfP220
 PoTiBGr136
 PoTiBGr409
 PoTiBGr143
 PoTiBGr211
 PoTiBGr209
 PoTiBGr213
 PoTimfP364
 PoTimfP147
 PoTimfJ50
 PoTimfJ2
 PoHL1
 PoTiBL37
0.58
0.79
0.84
//
 B31
0.81
0.96
Figure S8
0.05
