# Supplementary material for: Fine-Scale Phylogeographic Structure of Borrelia lusitaniae Revealed by Multilocus Sequence Typing
Source: PLoS One. 2008 Dec 23;3(12):e4002. doi: 10.1371/journal.pone.0004002 (PMC2602731; doi:10.1371/journal.pone.0004002)
Supplement: Figure S9 — Bayesian phylogenetic inference for ospA of B. lusitaniae, including samples from Italy and the Portuguese strains Poti B1-3. The figure shows that the Portuguese human isolate PoHL1 clusters with Italian samples (ITAh01, ITAh02; ‘European’ lineage), whereas samples from Mafra and Grândola cluster together with strains PotiB1-3 (‘African’ lineage (32)). Using MLST the Portuguese human isolate PoHL1 clusters with samples from Mafra. (0.07 MB PPT) [file pone.0004002.s009.ppt]

## Slide 1
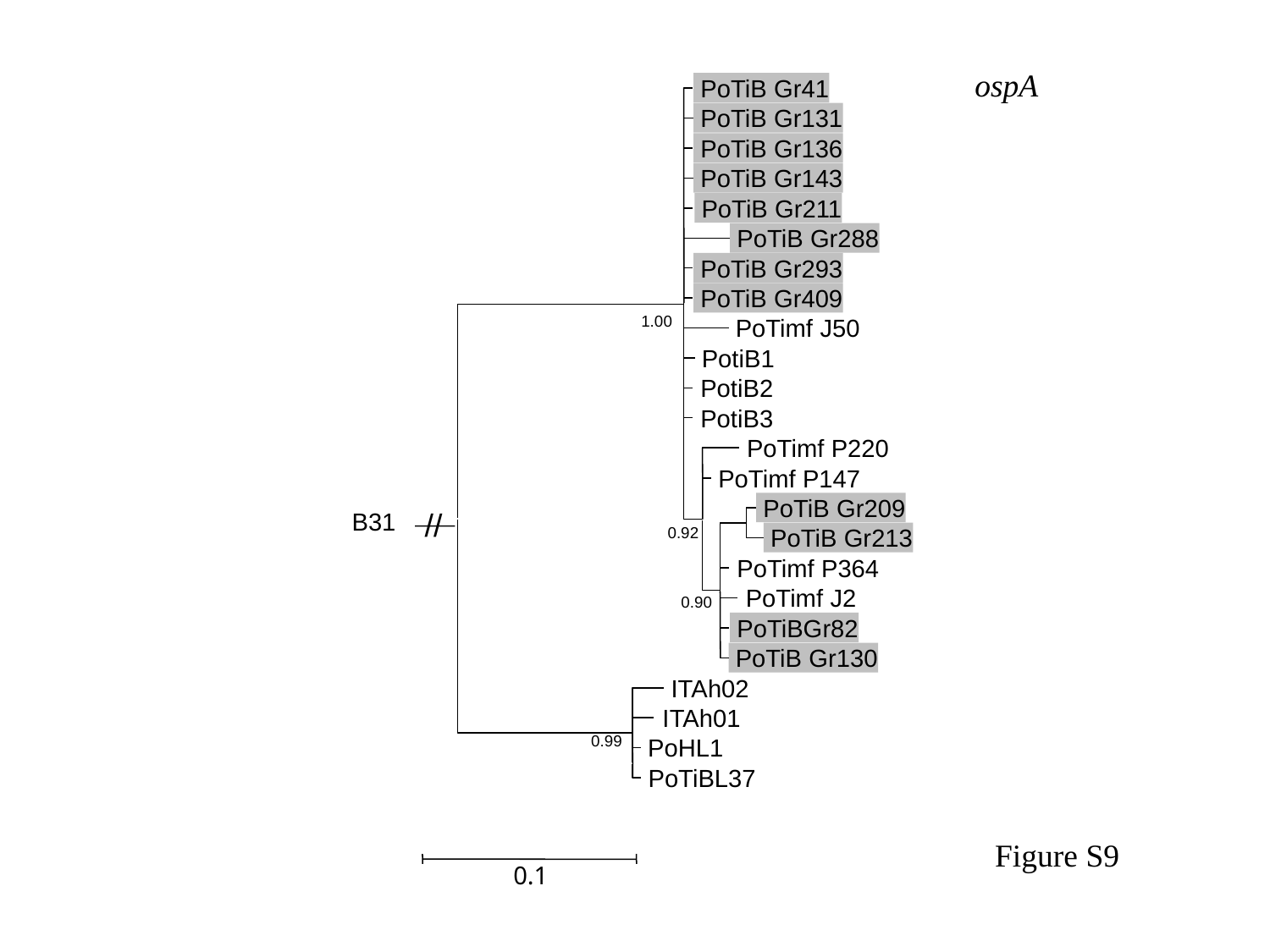

ospA
 PoTiB Gr41
 PoTiB Gr131
 PoTiB Gr136
 PoTiB Gr143
 PoTiB Gr211
 PoTiB Gr288
 PoTiB Gr293
 PoTiB Gr409
1.00
 PoTimf J50
 PotiB1
 PotiB2
 PotiB3
 PoTimf P220
 PoTimf P147
 PoTiB Gr209
//
 B31
0.92
 PoTiB Gr213
 PoTimf P364
 PoTimf J2
0.90
 PoTiBGr82
 PoTiB Gr130
 ITAh02
 ITAh01
0.99
 PoHL1
 PoTiBL37
Figure S9
0.1
