# Supplementary material for: Fine-Scale Phylogeographic Structure of Borrelia lusitaniae Revealed by Multilocus Sequence Typing
Source: PLoS One. 2008 Dec 23;3(12):e4002. doi: 10.1371/journal.pone.0004002 (PMC2602731; doi:10.1371/journal.pone.0004002)
Supplement: Figure S12 — Probability plots for recombination. Each diagram corresponds to likely substitution/recombination events inferred on the branches above nodes C to L in Figure S11 (e.g. events on the branch above node C). For nodes A and B no diagram was obtained. The columns in each diagram correspond to each of the seven housekeeping gene fragments. The scale on the y axis ranging from 0 to 1 refers to the probability of recombination. The height of the red lines represents the inferred probability for recombination. Only for clpX on the branch above node D a recombination event was inferred. The black crosses indicate inferred substitutions, their intensity being proportional to their probability. (0.25 MB PPT) [file pone.0004002.s012.ppt]

## Slide 1
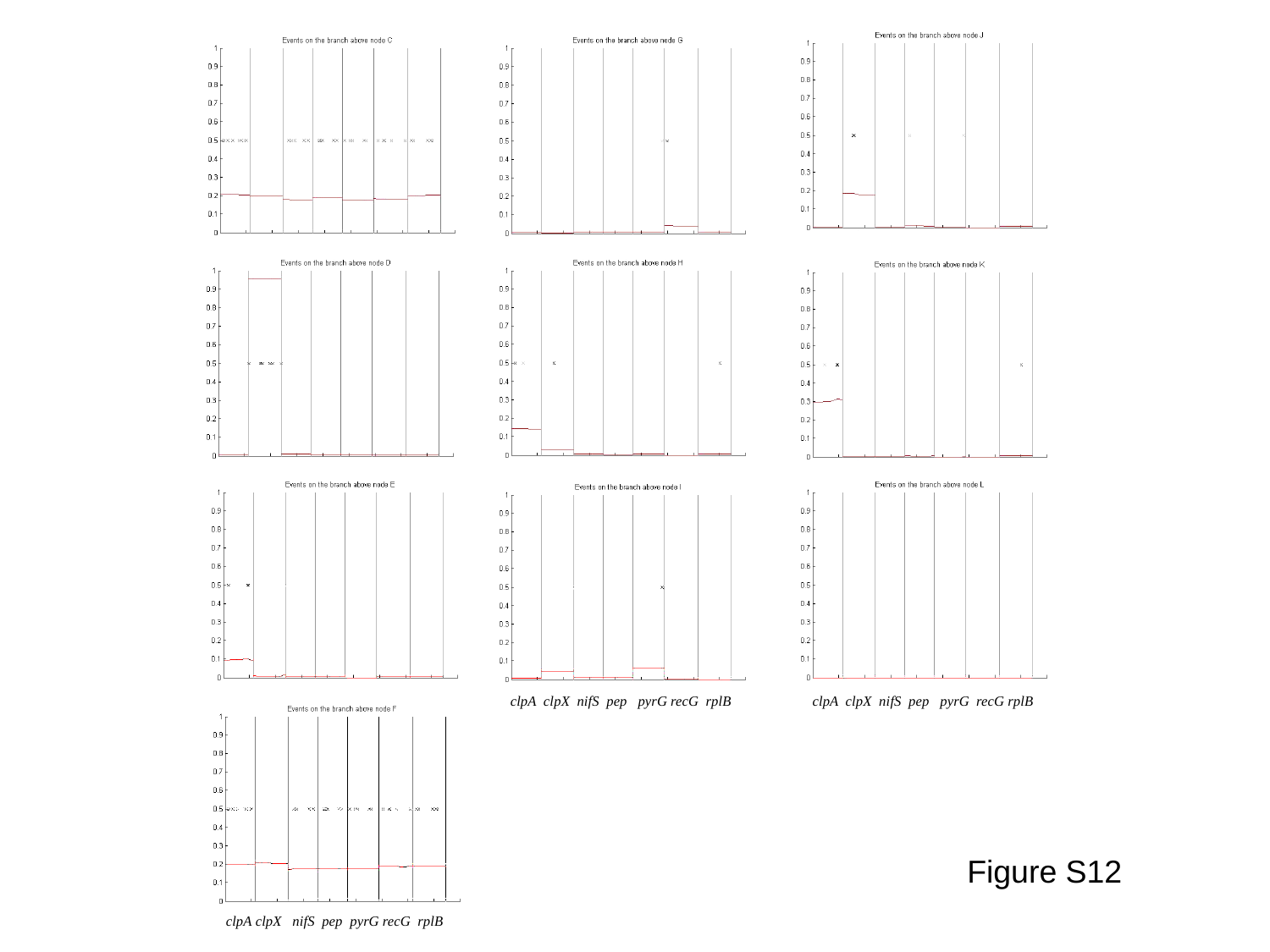

clpA clpX nifS pep pyrG recG rplB
clpA clpX nifS pep pyrG recG rplB
Figure S12
clpA clpX nifS pep pyrG recG rplB
